# The stiffening of the cell walls observed during physiological softening of pears

**DOI:** 10.1007/s00425-015-2423-0

**Published:** 2015-10-26

**Authors:** Artur Zdunek, Arkadiusz Kozioł, Justyna Cybulska, Małgorzata Lekka, Piotr M. Pieczywek

**Affiliations:** Institute of Agrophysics, Polish Academy of Sciences, Doświadczalna 4, 20-290 Lublin, Poland; The Henryk Niewodniczański Institute of Nuclear Physics, Polish Academy of Sciences, Radzikowskiego 152, 31-342 Kraków, Poland

**Keywords:** Atomic force microscopy (AFM), Cell wall, Firmness, Pear, Stiffness, Young’s modulus

## Abstract

**The Young’s modulus of the primary cell walls of pears decreases linearly during the pre-harvest on-tree maturation and increases during postharvest storage, and does not correlate with firmness of fruit.**

The determination of mechanical properties of cell walls is indispensable for understanding the mechanism of physiological softening and deterioration of quality of fruits during postharvest storage. The Young’s modulus of the primary cell walls from pear fruit (*Pyrus communis* L., cultivars ‘Conference’ and ‘Xenia’) during pre-harvest maturation and postharvest storage in an ambient atmosphere at 2 °C followed by shelf life was studied using atomic force microscopy (AFM). The results were related to the firmness of fruits, galacturonic acid content in water, chelator, sodium carbonate and insoluble pectin fractions, polygalacturonase and pectin methylesterase activities. The Young’s modulus of the primary cell walls decreased linearly during the last month of pre-harvest maturation from 3.2 ± 1.8 to 1.1 ± 0.7 MPa for ‘Conference’ and from 1.9 ± 1.2 to 0.2 ± 0.1 MPa for ‘Xenia’ which correlated with linear firmness decrease. During postharvest storage the cell wall Young’s modulus increased while firmness continued to decrease. Correlation analysis for the entire period of the experiment showed a lack of straightforward relation between the Young’s modulus of primary cell walls and fruit firmness. The Young’s modulus of cell walls correlated negatively either with galacturonic acid content in sodium carbonate soluble pectin (‘Conference’) or with insoluble pectin fractions (‘Xenia’) and positively with polygalacturonase activity. It was therefore evidenced that covalently linked pectins play the key role for the stiffness of fruit cell walls. Based on the obtained results, the model explaining the fruit transition from firm and crispy to soft and mealy was proposed.

**Electronic supplementary material:**

The online version of this article (doi:10.1007/s00425-015-2423-0) contains supplementary material, which is available to authorized users.

## Introduction

Cell walls determine macroscopic mechanical properties of fruit (Jarvis 2011; Cybulska et al. 2013; Gwanpua et al. 2014), as well as water transport and shrinkage (Fanta et al. 2014). Thus, the cell wall stiffness is a key parameter which must be considered to understand the mechanism of fruit softening. Stiffness (quantitatively described by the Young’s or elasticity modulus) is one of the most important parameters in microstructure-based models used for the prediction of macroscopic properties of plants (Fanta et al. 2014; Pieczywek and Zdunek 2014). Material properties of cell walls in plants change during growth and development due to biosynthesis and degradation of its constituents (Albersheim et al. 2011). It is also true for climacteric fruit where cell walls undergo substantial biochemical changes during on tree and postharvest maturation (Brummell and Harpster 2001). Although it is generally believed that during fruit ripening, the cell walls loosen and become weaker, neither the structural bases of these changes (Vicente et al. 2007) nor experimental evidences have been provided, so far. This is largely due to problems with the evaluation of cell wall structure and mechanical properties in conditions close to natural ones.

Measurements of cell wall elastic properties are difficult due to their small physical dimensions on the micrometer scale. So far, only a few methods have been developed to estimate mechanical properties of cell wall that may be applied to fruit. However, these developments focused on the estimation of cell wall properties from intact cells. In the micro-compression test, an individual living cell was compressed between two plates and a resulting force–displacement curve together with a computational model allowed for elucidation of properties of the cell wall (Mashmoushy et al. 1998; Shiu et al. 1999; Thomas et al. 2000; Blewett et al. 2000). A micro-penetration test was also applied for cell wall studies on intact tissue. Penetration of tissue was carried out using a parallel-sided probe with a diameter of about 15 % of a cell size (Hiller et al. 1996) and the deformation of a cell wall was simulated by a membrane analytical model which allowed to estimate cell wall stiffness (Davies et al. 1998). Micro-indentation is a similar technique to micro-penetration however uses smaller deformations (Routier-Kierzkowska and Smith 2013). It is performed by a flat or rounded indenter, a few micrometers in diameter (1–5 µm). Typically, the indentation depth is comparable to or larger than the cell wall thickness and the force is in a range of 1–100 µN. A device that allows the automation of micro-indentation measurements is the cellular force microscope (CFM) (Routier-Kierzkowska et al. 2012).

An atomic force microscope (AFM) has been applied for nano-indentation of biological materials (Radmacher et al. 1994, 1995; Kuznetsova et al. 2007; Lekka and Laidler 2009; Lekka 2012; Lekka et al. 2012; Kurland et al. 2012). The technique combines imaging of surface topography with a nanometer resolution and sensing of the nano-mechanical properties of the sample. The Young’s modulus for each sample has been determined by fitting a mathematical model describing the contact mechanics between the AFM tip and sample. The Hertz–Sneddon model is the most commonly used one, under the assumption that a sample is a linearly elastic and isotropic solid with thickness infinitely extending to a half space (Sneddon 1965). The AFM has been recently applied to measure mechanical properties of plant cells: suspended grapevine cells grown in liquid medium (Lesniewska et al. 2004), the primary cell wall of shoot apical meristems (Milani et al. 2011, Peaucelle et al. 2011), rosette leaves (Hayot et al. 2012) and epidermal cells of living roots of *Arabidopsis thaliana* (Fernandes et al. 2012), and suspended cells extracted from tomato pericarp (Zdunek and Kurenda 2013). However, the method has not yet been applied to evaluate the changes of cell wall stiffness during fruit maturation.

The evaluation of the Young’s modulus of the wall in intact plant cells, regardless of the method used, requires the assumptions about turgor pressure and cell wall thickness that are usually difficult to estimate. This difficulty and the problem of small dimensions of natural cell walls has been partially solved by performing mechanical tests on model membranes composed of bacterial cellulose, pectins and xyloglucan (Cybulska et al. 2010, 2011). The material composition of these membranes is similar to the natural cell walls and may be considered as a representative system to simulate various effects. However, the artificial materials are not able to mimic complex biochemical processes occurring in fruit cell walls during maturation.

In our work, the procedure for stiffness measurements of cell walls extracted from fruit with the use of the AFM was elaborated. To avoid problems with the turgor and cell wall thickness, measurements were performed on cell wall fragments prepared as alcohol insoluble residues after tissue crushing. The cell walls were studied in deionized water, thus in a hydrated state that mimics the conditions close to natural ones. The goal of our studies was to evaluate the Young’s modulus of the cell walls as a function of maturation time, including pre-harvest development (i.e., fruit on trees) and postharvest storage of two pear (*Pyrus communis* L.) cultivars ‘Xenia’ and ‘Conference’. Such an approach enabled us to investigate the relation between cell wall stiffness and macroscopic firmness of fruit. Changes in mechanical properties of cell walls were interpreted based on their biochemical characteristics, i.e., the presence of galacturonic acid in pectin fractions, polygalacturonase and pectin methylesterase activities. The obtained results provided an important contribution into a structure-based model of fruit softening.

## Materials and methods

### Fruits

Pear (*Pyrus communis* L.) fruits of two cultivars ‘Conference’ and ‘Xenia’ from the same orchard were used in our studies. Pears were picked at five pre-harvest stages within 27 and 34 days before harvest for ‘Conference’ and ‘Xenia’, respectively. In this period, pears were already fully expanded. Pears harvested at the optimum time were stored in a cold room at 2 °C and RH ~80–90 % in ambient atmosphere for 120–145 days for ‘Conference’ and ‘Xenia’, respectively. During this period, the material was studied at five stages for ‘Conference’ and four stages for ‘Xenia’ with an interval of ~30 days. Each stage was followed by 3–7 days of shelf life at 20 °C and RH ~40–50 % to stimulate softening. In total, the experiment consisted of 15 stages for each cultivar. Each batch of pears consisted of at least ten fruits of similar size without visible damages. These pears were used first for firmness determination and then smashed for collection of cell wall material and for other biochemical analyses.

### Firmness

Firmness of individual pears without skin was measured using a universal testing machine (Lloyd LRX, Lloyd Instruments Ltd., Hampshire, UK) in the puncture test with a probe of 11.1 mm. A crosshead speed was set to 20 mm/min and maximum penetration depth was 8 mm. Firmness was defined as the maximum force value observed in a force-penetration curve.

### Cell wall material (CWM)

Cell wall material (CWM) was isolated from parenchyma tissue as alcohol insoluble residue (AIR), (Renard 2005). 20 g of fruit pulp was boiled with 70 ml of 70 % ethanol for 20 min. The sample was chilled, filtered using a nylon filter and mixed with 30 ml of 70 % ethanol. After filtration and a negative result from the phenol–sulfuric acid assay for the presence of sugars (Dubois et al. 1956), the sample was washed twice with 10 ml of 96 % ethanol and 50 ml acetone and dried at 40 °C.

### Pectin fractions

Pectins were isolated during sequential extraction according to the method proposed by Redgwell et al. (1988) with some modifications. AIR was stirred in deionized water for 6 h at 20 °C and then centrifuged. The supernatant was collected as the WSP fraction, whereas the residue was mixed with 0.1 M cyclohexane-*trans*-1,2-diamine tetra-acetate (CDTA) (pH 6.5) and stirred at 25 °C for 6 h, filtered and again stirred with CDTA for 2 h. The supernatant was separated as the CSP fraction and the residue was diluted in 0.05 M sodium carbonate (Na_2_CO_3_) and 20 mM sodium borohydride (NaBH_4_) was added. This solution was then stirred for approximately 20 h at 1 °C, filtered and again stirred in the same solvent for 2 h at 20 °C. The DASP fraction was collected after centrifugation as a supernatant and a residue was collected to determine the GalA content in the insoluble pectin fraction.

### Galacturonic acid (GalA) content

Galacturonic acid (GalA) in pectic fraction contents was determined using the San^++^ Continuous Flow Analyzer (Skalar, Breda, The Netherlands) according to the colorimetric method by Blumenkrantz and Asboe-Hansen (1973). The sample was totally decomposed in 96 % sulfuric acid (H_2_SO_4_) with di-sodium tetra borate Na_2_B_4_O_7_^.^10H_2_O. Then the products were transformed into furfuric derivatives. The derivatives reacted with the 3-phenyl phenol to form a colored dye, which was measured at 530 nm. Galacturonic acid solutions (10–100 μg/ml) were used as standards. GalA content in pectin fractions was expressed in microgram per milligram of AIR.

### Polygalacturonase (PG) and pectin methylesterase (PME) enzymatic activity

Enzymatic activity of pectinases was determined according to the method described by Wei et al. (2010) with some modifications. Briefly, enzymes of a cell wall were extracted from frozen fruit pulp. Powdered 3 g flesh was stirred into 6 ml of cold 12 % polyethyleneglycol containing 0.2 % sodium bisulite and centrifuged for 10 min at 6000*g*. The pellet was washed with 0.2 % sodium bisulfite at 4 °C. Next the pellet was extracted with 6 ml of cold extraction buffer containing 1 M sodium acetate (pH 5.2), 1 M NaCl, 2 % (v/v)-mercaptoethanol, and 5 % (w/v) polyvinylpyrrolidone (PVP), at 4 °C for 1 h. The homogenate was centrifuged for 10 min at 6000*g*, and the supernatant was used to assay for enzyme activity.

Polygalacturonase (PG) activity was determined in the following way: enzyme extract (0.2 ml) was mixed with 0.8 ml of 0.5 % polygalacturonic acid in 50 mM sodium acetate buffer (pH 5.2), and incubated at 37 °C for 2 h. Next, 2 ml of borate buffer (0.1 M, pH 9.0) and 0.3 ml of cyanoacetamide were added to the reaction mixture. After inactivation of the enzymes by boiling for 10 min and then cooling, absorbance was read at 320 nm. GalA was used as standard. One unit of activity was defined as 1 µg of GalA released from gram fresh weight (FW) per minute.

To determine the activity of pectin methylesterase (PME), 1 ml of crude extract was mixed with 4 ml of 1 % (w/v) citrus pectin and titrated with 0.01 M NaOH to maintain pH 7.4 while incubating at 37 °C for 1 h. One unit of activity was calculated as 1 µmol NaOH consumed by gram FW per minute.

### Cell wall stiffness

**Fig. 1:**
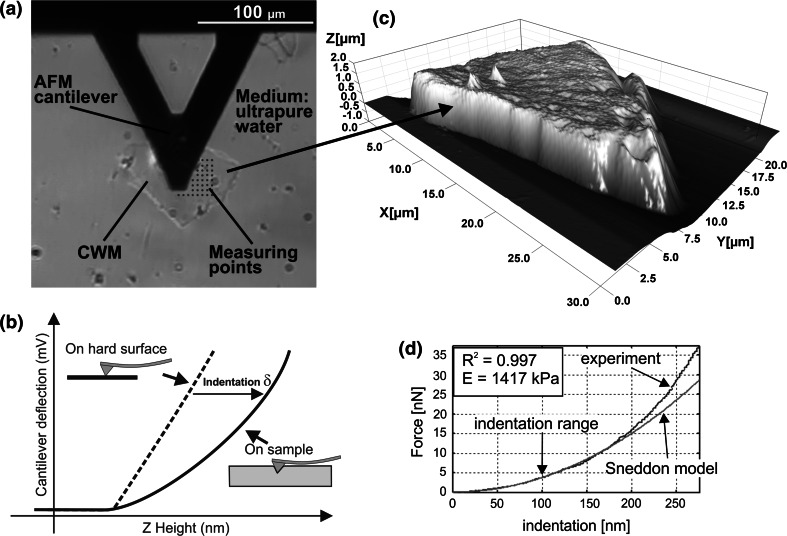
The idea of the Young’s modulus *E* estimation for cell walls. **a** *Top view image* showing the AFM cantilever and a fragment of the cell wall material (CWM) in water. *Dots* denote a grid of points set for the force curve collection. **b** Schematic presentation of how the indentation is determined on a soft sample*. Dashed line* is sensitivity calibration curve recorded on a glass slide (an infinite hard surface). **c** Example of 3D topography of CWM used in experiment which showed that thickness of cell walls in pears is about 1 µm. **d** Screenshot from the proprietary code used to fit the Hertz–Sneddon model to the experimental data within the indentation depths of 0–100 nm. The goodness of the fit *R* ^*2*^ > 0.8 was set to be an acceptance threshold for the fitting quality

CWM suspension (1 mg/ml) was dropped on a microscope glass slide and then air-dried. Ten minutes before tests, ultrapure water (Milipore) was added to swell samples (Fig. 1a).

Atomic force microscope (AFM) Bioscope Catalyst II equipped with Nanoscope V controller (Bruker, Billerica, MA, USA) was working in an indentation-type mode (Loparic et al. 2010). For indentation, a silicon nitride cantilever SNL-10 (Bruker) with a nominal spring constant *k*_*n*_ = 0.35 N/m and a resonance frequency *ω*_*n*_ = 65 kHz was chosen. A mean opening angle of the tip *α* = 20.8° ± 5.2° was calculated from the front, back and side angles. The measured resonance frequency of thermally excited cantilevers in air was *ω* = 57.8 ± 1.0 kHz. With the assumption that the actual mass of cantilever is equal to that of a nominal one, the spring constant was calculated from the proportion *k*/*k*_*n*_ = *ω*^2^/*ω*_*n*_^2^. The obtained value of the cantilever spring constant was *k* = 0.28 ± 0.01 N/m. Deflection sensitivity (nm/V) was determined from measurements carried out on glass substrate in aqueous conditions (Fig. 1b). At least five repetitions were made for both calibration steps. The force *F* (in nN) was calculated using Hooke’s law *F* = *kx*, where *x* is the cantilever deflection (in nm).

For the each stage of the experiment, ten randomly chosen fragments of CWM were tested. For the each CWM, a scan of 10 µm × 10 µm was recorded and then 64 force curves with an approach rate of 1 µm/s were collected in a regular grid of 8 × 8 points: the distance between points was 1.43 µm (Fig. 1a). The Young’s modulus *E* was calculated for each individual force curve within the indentation range 0–100 nm by fitting the Hertz–Sneddon model (Fig. 1d):

1 $$F = \frac{E}{{1 - \upsilon^{2} }}\frac{2\tan \alpha }{\pi }\delta^{2}$$

where *F* is the force, *δ* is indentation, *α* is the tip opening angle and *ν* is the Poisson ratio. The Poisson ratio of 0.3 (adequate for polymers) was chosen. The indentation up to 100 nm was less than 10 % of the sample height (about 1 µm) estimated from several scans, as example presented in Fig. 1c. The indentation depth applied was much lower than the height of the tips (2.5–8 µm) as provided by the manufacturer. The contact point between the AFM tip and sample surface was estimated manually in each force curve as the point when the force started to deviate from a base line. Fitting of the model to experimental curves was performed using a proprietary code developed in Matlab (MathWorks, Natick, MA, USA). The fitting quality was verified using a Matlab’ *R*-square (*R*^*2*^) statistic measure that is the square of the correlation between the experimental values and the predicted by model values. It has been decided arbitrarily after analysis of all curves (640 curves for each stage) that if the goodness of the fit *R*^2^ was lower than 0.8 a force curve could not be fitted by the Hertz–Sneddon model and was removed from further analysis. The mean value of the Young’s modulus and standard error were calculated from the remaining 400–600 curves for each stage.

### Statistical analysis

A significant difference between means was verified using a one-way ANOVA statistical test, followed by post hoc Tukey’s honestly significant difference test (Statistica 10, StatSoft, Inc., Tulsa, USA). The obtained Young’s modulus values were presented as mean values with a standard error.

## Results

### Firmness

**Fig. 2:**
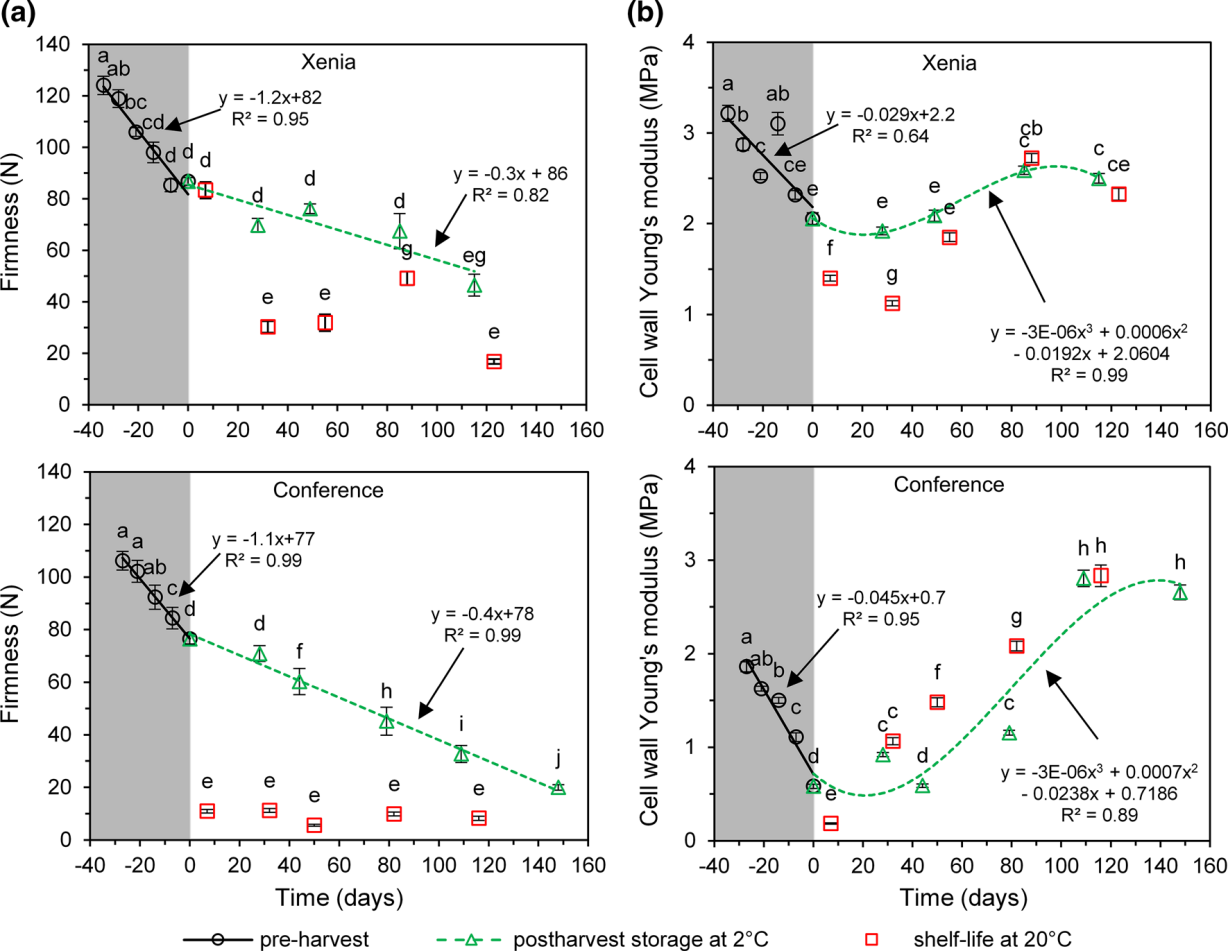
Firmness (**a**) and cell wall Young’s modulus (**b**) changes for fruit collected during pre-harvest maturation (shadowed region, *open circles*) and during postharvest storage in a cold room at 2 °C and RH ~80–90 % in ambient atmosphere (*green triangles*). Time zero means the harvest time. *Squares* present shelf life points after predated storage in a cold room. *Error bars* are standard errors. The same letters mean no significant difference (*P* < 0.05)

Changes in firmness of pears during pre-harvest maturation and postharvest storage are presented in Fig. 2a. In the pre-harvest period, firmness was linearly decreasing with the rate of about 1 N per day. For ‘Xenia’ pear, its value dropped from 124 ± 12 N (−34 days) to 87 ± 5 N at harvest time. For ‘Conference’ pear, a similar decrease was observed, i.e., from 106 ± 11 N (−27 days) to 76 ± 6 N at harvest time. Such a trend is qualitatively in agreement with the previous studies carried out by Murayama et al. (1998) on pears. In cold storage at 2 °C in an ambient atmosphere, fruit continued to linearly decrease its firmness although with lower rate which was about 0.3–0.4 N per day. In the final stage of the experiment, ‘Xenia’ pear softened to 46 ± 13 N while ‘Conference’ pear to 20 ± 3 N. The postharvest softening is typically observed for climacteric fruit (Murayama et al. 1998, 2002), however, the rate depends on the storage method (Gwanpua et al. 2014). Cold storage in a natural atmosphere causes more rapid decrease of firmness, compared to other methods (not studied here) due to relatively high oxygen levels which helps to accelerate ripening by increasing the rate of oxidative breakdown reactions. Few days of shelf life at 20 °C, that followed the cold storage stages, caused accelerated deterioration of pear firmness (squares in Fig. 2a). In the case of ‘Conference’ pear, shelf life caused the decrease of firmness down to ~10 N in all shelf life points whereas for ‘Xenia’ pear, most of the shelf life cases significantly decreased firmness to about 16–50 N with much larger variability as compared to the second cultivar. It should be underlined that for one event just 7 days after the harvest, the decrease was not significant in the relation to the predated point.

### The Young’s modulus of pear cell walls

**Fig. 3:**
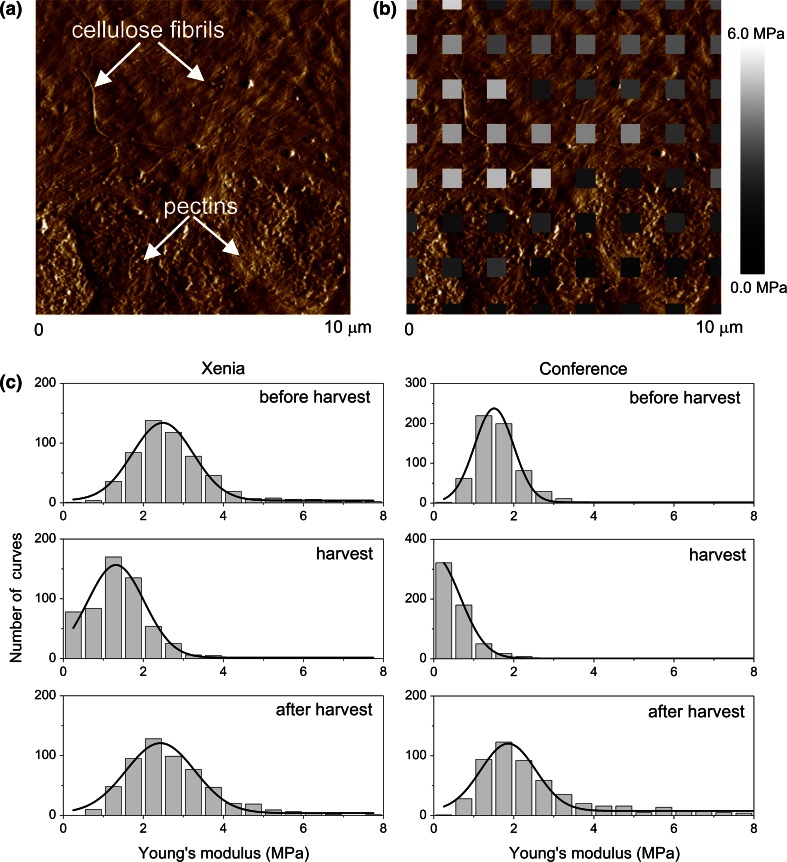
The Young’s modulus spatial variability in cell walls obtained for pear. **a** Typical AFM image of cell wall material (CWM) from a pear (error mode). Two regions are observed. In the* upper part* of the image cellulose fibrils are clearly visible, while in the* bottom part* of the image the fibrils are probably covered by the pectins’ matrix. **b** The Young’s modulus map imposed on the surface image. The distance between centers of the *grey squares* is 1.4 μm. The various *grey colors* denote spatial variability of the estimated Young’s modulus. **c** The exemplary distributions of the Young’s modulus obtained for three distinct experimental stages: before harvest (20 days before harvest), at harvest and after harvest (about 80 days after harvest)

Figure 3a presents an example of the cell wall image (so-called “error image”) depicting regions of fibrils embedded in amorphous matrix whereas Fig. 3b shows the corresponding map of stiffness. In our experiments, the cantilevers with sharp tips were used which ends up in a comparable diameter with the fibrils one. In such a way, a spatial variability in sample stiffness can be probed since such a sharp tip indents either a single fibril or space between neighbouring fibrils. Figure 3a shows the region (the bottom part of the image area) where fibrils are apparently covered by a matrix of pectins. Its presence leads to smaller local Young’s modulus depicted on the stiffness map (Fig. 3b). Figure 3c presents histograms of the Young’s modulus for distant experimental stages for ten CWM fragments considered to characterize the stages. Despite large standard deviations, a clear shift of histograms for the harvest stage is visible compared to the before and after harvest ones. Therefore, to follow the time changes of the Young’s modulus of cell walls, the mean value was used and a significance of the effect was checked by ANOVA post hoc analysis (at *P* < 0.05).

The relations of the Young’s modulus as a function of time, determined for the pear cell wall, are shown in Fig. 2b. The profile of changes was very similar for both cultivars but surprisingly it was different from the firmness changes presented in Fig. 2a. In the pre-harvest period, the Young’s modulus of the cell wall linearly decreased from 3.2 ± 1.8 to 2.0 ± 1.5 MPa and from 1.9 ± 1.2 to 0.6 ± 0.5 MPa for ‘Xenia’ and for ‘Conference’, respectively. The rate of decrease was about 30 kPa per day for ‘Xenia’ and 45 kPa per day for ‘Conference’. The lowest stiffness of the cell wall was noted just after the harvest and it was 1.1 ± 0.7 and 0.2 ± 0.1 MPa for ‘Xenia’ and ‘Conference’, correspondingly. For samples stored longer than 40 days in cold storage in an ambient atmosphere, the cell wall Young’s modulus started to increase significantly. At the end of the cold storage, i.e., after around 120 days, the change of Young’s modulus seemed to be inhibited, ending at the modulus values of about 2.5 and 2.8 MPa for ‘Xenia’ and for ‘Conference’, respectively. The variations in the modulus values during postharvest cold storage for both cultivars had similar characters that could be described by a third-order polynomial. However, the amplitude of changes was different—it was much larger for the ‘Conference’ pear.

The effect of shelf life on cell wall stiffness showed cultivar-dependent behaviour. For ‘Conference’, shelf life caused in most cases an increase of the cell wall Young’s modulus compared to predated cold storage while for ‘Xenia’ the effect was opposite, i.e., shelf life caused the decrease of cell wall stiffness. From Fig. 2b, it is worth to underline that in the pre-harvest period and also during about first 3 months of postharvest period, the cell walls in ‘Xenia’ were stiffer than in ‘Conference’ pears.

## Discussion

In our studies, the Young’s modulus of cell walls collected from pears, determined from the AFM measurements, is generally lower as compared to previously reported values for other plants, like tomato, apple or potato. For tomato suspension cells, the Young’s modulus of cell walls estimated by microcompression technique was in the range of 0.1–2.3 GPa (Blewett et al. 2000). The maximum value (of 2.3 GPa) was then used for a computational model of compression of single tomato suspension cells from a root radicle callus (Dintwa et al. 2011). Another study has reported the elastic modulus of the cell walls collected from pericarp of commercially grown tomatoes, also estimated using micro-compression technique, was within the range of 30–80 MPa (Wang et al. 2006). For apples, for the purpose of a cell deformation model, the Young’s modulus of cell walls of 26.4 and 52.8 MPa was taken. For potatoes, the estimated Young’s modulus of cell walls from micro-penetration measurements was of 105 MPa (Davis et al. 1998). Apart from the different commodities compared above, the main difference stems from the applied methodology of the measurements of cell wall stiffness. In most techniques reported above, measurements are carried out on a much larger area as compared to the AFM measurements. Therefore, the obtained Young’s modulus originates from a large volume of the sample. In the AFM, the average value is a sum of all measurements carried out in nanoscale. Moreover, in the previous methods the intact cells were studied and their deformations were close or over a strength of the cell wall that probably led to its higher stiffness compared to results obtained by low AFM indentation. It should be noted also that in the previous methods some uncertainty of estimation may have come from the assumptions about turgor or cell wall thickness which is avoided in the AFM measurements.

Similar values to the cell walls stiffness of pears observed in our studies were obtained by the AFM indentation of suspension-cultured cells of *Arabidopsis thaliana* (Radotić et al. 2012). For the indentation 80 nm stiffness of cell walls was estimated to be in the range of 0.1–1.0 MPa, depending on the age of the growing *Arabidopsis* cells.

### Why does the cell wall stiffness not correlate with fruit firmness?

The correlation matrix built from data of all experimental stages is shown in Table 1. The correlation analysis confirmed, as observed in Fig. 2a and b, a lack of straightforward correlation between firmness and the Young’s modulus of cell walls. For the entire studied period, the positive and significant (*P* < 0.05) correlation was found for ‘Xenia’ pear while for ‘Conference’ one the correlation was slightly negative and not significant (Table 1). An unambiguous and strong positive relationship between firmness and the Young’s modulus for both cultivars could be found only in the pre-harvest period (Fig. 2a and b). In the postharvest period, the relation between these variables is opposite; while firmness was continuously diminishing, cell wall stiffness was increasing. Moreover, the decrease of firmness in shelf life conditions does not clearly reflect in the cell wall elasticity.

**Table 1:** Correlation matrix among variables studied for pear cv. ‘Conference’ and ‘Xenia’

	Firmness	GalA in WSP	GalA in CSP	GalA in DASP	GalA in insoluble	PG	PME
Conference							
Cell wall Young’s modulus	−0.23	0.14	−0.04	−0.69*	0.36	0.65*	−0.28
Firmness	1.00	−0.74	0.00	0.59*	−0.42	−0.28	0.36
GalA in WSP		1.00	0.29	−0.57*	0.27	0.09	−0.48
GalA in CSP			1.00	0.14	−0.44	−0.10	0.10
GalA in DASP				1.00	−0.39	−0.44*	0.51
GalA in insoluble					1.0	0.46	−0.54*
PG						1.00	−0.68*
PME							1.00
Xenia							
Cell wall Young’s modulus	0.52*	−0.30	−0.16	0.25	−0.56*	0.66*	0.68*
Firmness	1.00	−0.84*	−0.43	0.88*	−0.69*	0.46	0.81*
GalA in WSP		1.00	0.72*	−0.79*	0.41	−0.35	−0.63*
GalA in CSP			1.00	−0.38	−0.02	−0.24	−0.32
GalA in DASP				1.00	−0.47	0.14	0.55*
GalA in insoluble					1.00	−0.55*	−0.63*
PG						1.00	0.60*
PME							1.00

Table shows Pearson’s correlation coefficients of linear regression between variables (*n* = 15)

*GalA* galacturonic acid, *WSP* water soluble pectins, *CSP* chelator (CDTA) soluble pectins, *DASP* sodium carbonate soluble pectins, *PG* polygalacturonase activity, *PME* pectin methylesterase activity

* Significant correlation (*P* < 0.05)

The first reason for the lack of straightforward relation of cell wall stiffness with firmness obviously comes from distinct scales of these parameters and multiple factors contributing to tissue softening. Firmness is the macroscopic parameter of tissue and it is related to the properties of several building blocks at different length scales, i.e., cell walls, middle lamella (Jarvis et al. 2003), cell size (Cybulska et al. 2012) and turgor (Vicente et al. 2007). A decline in firmness coincides with multiple coordinated processes, including dissolution of the middle lamella and solubilization of hemicellulose and pectin cell wall polysaccharides (Brummell and Harpster 2001), and turgor loss (Saladie et al. 2007). The decline in turgor causes wall relaxation that presumably is one of the reasons of changes in cell wall architecture, reduction in intercellular adhesion that results in increase of intercellular spaces and eases water transpiration (Niklas 1992; Saladie et al. 2007). Therefore the role of cell wall stiffness in firmness of fruit may be overshadowed by other components of the microscopic biomechanical model of fruit tissue as turgor and intercellular adhesion. Moreover, in the puncture test used for firmness evaluation, tissue and cell walls are deformed over an elastic limit and finally disrupted. Thus, the cell wall elasticity measured in the range of low deformations by AFM may not directly relate to the nonlinear large deformation by the puncture probe.

The second possible reason may refer to a different intrinsic course of changes in mechanical properties of middle lamella and primary cell wall occurring in the postharvest period. Postharvest modification of pectins in the middle lamella unquestionably softens the fruit because of the decrease of cell-to-cell integrity (Jarvis et al. 2003; Vicente et al. al. 2007; Ng et al. 2013). It is widely accepted that this process results in the decreasing crispness, juiciness, and increasing mealiness sense due to the change of the destruction mode from cell wall rupturing to cell-to-cell debonding (Harker and Hallett 1992; Harker et al. 1997a, b). In our studies, the AFM tip probed most likely the primary cell walls due to the boiling process applied in the protocol of the alcohol insoluble residue preparation which alters the cell–cell adhesion (Marry et al. 2000; Renard 2005).

Based on our findings, the model of the fruit transition from firm and crispy to soft and mealy in the postharvest period is proposed. At the harvest date and shortly after harvest the primary cell walls were the softest in both studied pear cultivars. Simultaneously, the integrity of middle lamella was presumably still relatively high as the result of low PG activity and calcium crosslinking of homogalacturonan. Such a combination of mechanical properties and high turgor inside cells causes better tissue integrity and favorable conditions for cracking through the cell walls. It makes pear tissue firm, juicy and crispy. Then, during storage, the primary cell walls become stiffer while middle lamella decays. Both the stiffening of cell walls and deterioration of middle lamella promote the destruction of tissue through cell-to-cell debonding. This leads to soft and mealy properties of fruits.

As shown in ESM_1, GalA in pectin fractions changed during the experiment. The cell wall Young’s modulus correlated negatively (*P* < 0.05) either with the content of GalA in DASP (‘Conference’) or with the GalA in the insoluble fractions (‘Xenia’) whereas in other pectin fractions such correlations were not significant (Table 1). Sodium carbonate extracts pectins covalently linked in cell wall thus the significant correlations suggest an important role of pectins strongly linked in cell walls for maintaining their mechanical properties. It may come from the unique self-assembly ability of DASP fraction observed in vitro on mica in previous studies (Cybulska et al. 2015; Zdunek et al. 2014). The structure of DASP on mica is formed as arranged in parallel, spaced and interlinked straight molecules. Although it is still not known how DASP molecules behave in natural conditions, such a gel-like structure, if existent in tissue as well, may loosen the cellulose/hemicellulose network and thereby decrease its stiffness. This is in line with a general concept of the role of pectins in cell wall assembly. They form hydrated gels that push microfibrils apart and ease their sideways slippage during cell growth (Cosgrove 2005). Moreover, it has been shown by Cybulska et al. (2015) that during postharvest storage of carrot, the network of DASP molecules on mica was lost. Therefore, one may conclude that the degradation of the covalently linked pectins is the reason of cell wall stiffening in the postharvest period.

PG and PME activities during studied period for two pear cultivars are shown in ESM_2. The correlation analysis (Table 1) showed a significant positive relationship of the Young’s modulus with the PG activity for both cultivars whereas PME correlated with the cell wall stiffness for ‘Xenia’ cultivar only. It has been previously reported that PG has a slight influence on fruit softening (Brummell and Harpster 2001). This agrees with insignificant and inconsistent correlations of PG with firmness for two pear cultivars observed in our studies (Table 1). On the other hand, a pronounced role of PG activity on the softening of pears was found by Ahmed and Labavitch (1980). A negative, but small, correlation of PG activity with firmness was observed for ‘Jonagold’ apples (Gwanpua et al. 2014). Our results show that larger cell wall stiffness is associated with higher enzymatic activity of PG (Table 1). PG activity correlated also negatively either with GalA content in DASP (‘Xenia’) or with GalA in insoluble pectins (‘Conference’). This is in agreement with the correlations of GalA with the cell wall Young’s modulus. It confirms the previous conclusion that PG mediated degradation of covalently linked pectins in the primary cell walls results in their larger stiffness.

The elucidated role of pectins in cell wall stiffness is also in line with studies on cell wall analogs containing bacterial cellulose, xyloglucan and pectins (Cybulska et al. 2010; Gu and Catchmark 2014). Such composites, studied in the tensile test, were stiffer with decreasing content of pectins.

## Conclusions

Our studies revealed that the Young’s modulus of the primary cell wall in pears decreases during pre-harvest maturation and increases when fruit continues softening during postharvest storage. This discrepancy during postharvest period may be due to degradation of pectins in middle lamella causes decreasing of a cell-to-cell adhesion whereas in the primary cell wall causes its stiffening. This is in line with the theory that decreasing crispness and increasing mealiness of fruit during postharvest storage relate to the change of the failure mode from cell wall rupturing to cell-to-cell debonding.

Based on our results and on previous work on cell wall analogs, we can conclude that pectins play a key role in changes of cell wall stiffness during pre- and postharvest periods. The stiffness of the primary cell walls largely depends on galacturonic acid content either in sodium carbonate soluble or in insoluble pectin fraction. The PG-mediated depolymerization of these pectins causes stiffening of the primary cell walls in pear fruit. This may be linked to previously found self-assembly of the sodium carbonate soluble pectins to a regular gel-like network which is degraded during maturation. This study elucidated a general role of pectin backbone made of galacturonic acid for the mechanical properties of cell walls; however deeper insight into polysaccharide structure is necessary to fully interpret the mechanism and relation of the pre- and postharvest fruit softening with the cell wall mechanical properties. Our studies have demonstrated that the AFM technique is an useful tool to evaluate stiffness of the cell wall. It opens a venue to study the role of other cell wall components considered as important for cell wall mechanics, like hemicelluloses, neutral sugars and related enzymes.

Our study showed that the Young’s modulus of cell wall in pear is in the range of few megapascals and this value is suggested for further computational models to predict mechanical properties of this fruit. However, it is important to consider the actual value of the cell wall Young’s modulus which depends on the maturation stage of fruit.

### *Author contribution statement*

AZ designed experiment, interpreted data and wrote the manuscript; AK performed AFM experiment; JC prepared CWM samples, did biochemistry, and discussed role of pectins in CWM mechanics; ML proposed method and discussed results on CWM stiffness evaluation; PMP prepared software for the Young’s modulus evaluation. All authors read and approved the manuscript.

## Electronic supplementary material

Supplementary material 1 (PDF 320 kb)

Supplementary material 2 (PDF 223 kb)

## Abbreviations

AFM: Atomic force microscopy
CWM: Cell wall material
DASP: Sodium carbonate soluble pectins
GalA: Galacturonic acid
PG: Polygalacturonase
PME: Pectin methylesterase
