# Supplementary material for: The stiffening of the cell walls observed during physiological softening of pears
Source: Planta. 2015 Oct 26;243:519–29. doi: 10.1007/s00425-015-2423-0 (PMC4722064; doi:10.1007/s00425-015-2423-0)
Supplement: Supplementary file 1 — Supplementary material 1 (PDF 320 kb) [file 425_2015_2423_MOESM1_ESM.pdf]

The stiffening of the cell walls observed during physiological softening of pears

Planta

Artur Zdunek[1], Arkadiusz Kozioł[1], Justyna Cybulska[1], Małgorzata Lekka[2], Piotr M.Pieczywek[1]*

[1]Institute of Agrophysics, Polish Academy of Sciences, Doświadczalna 4, 20-290 Lublin, Poland, [2]The Henryk Niewodniczański Institute of Nuclear Physics, Polish Academy of Sciences, Radzikowskiego 152, 31-342 Kraków, Poland, ***corresponding author** a.zdunek@ipan.lublin.pl

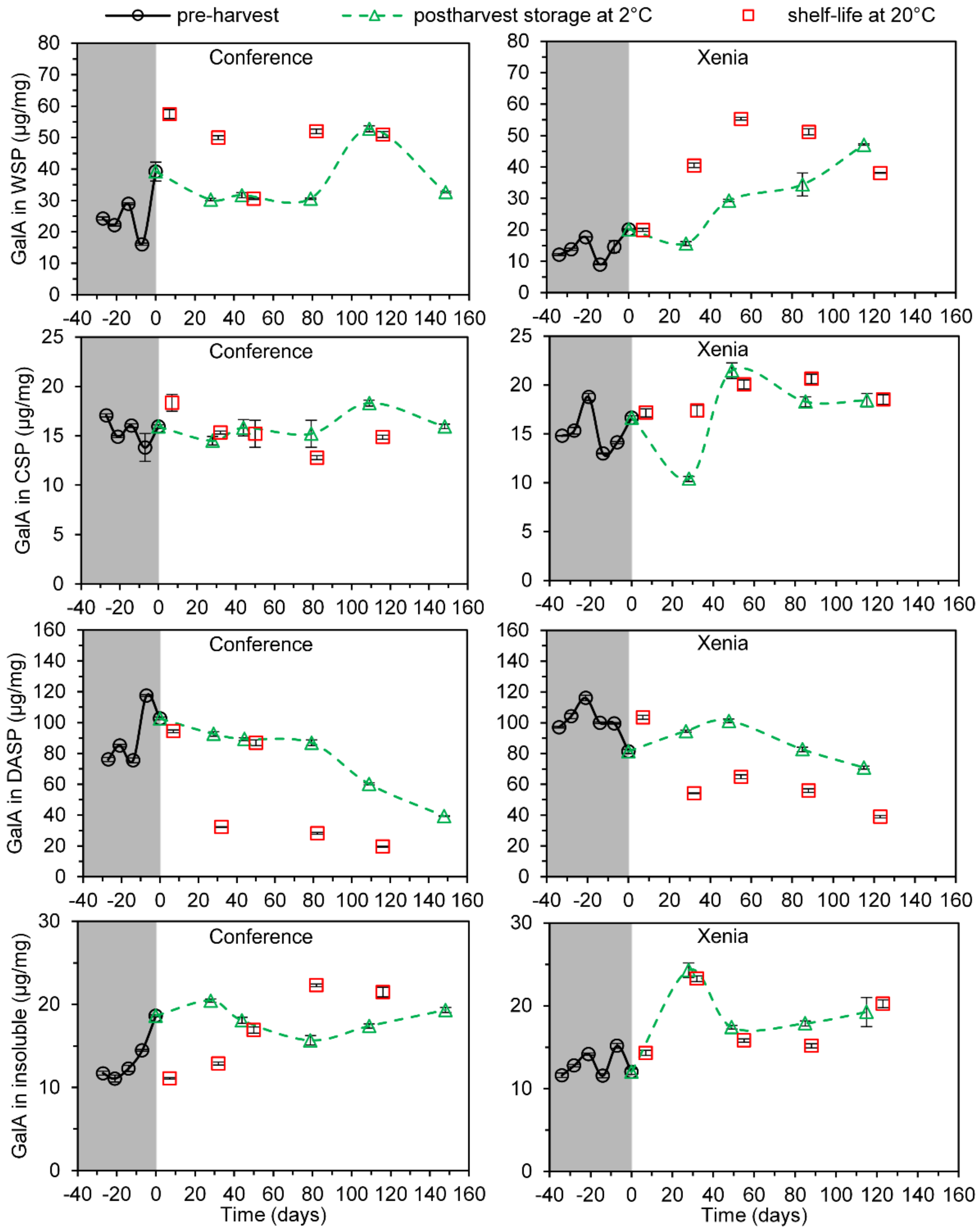

ESM_1. Changes of galacturonic acid (GalA) content in pectin fractions extracted from cell walls of pear cv. ‘*Conference*’ and ‘*Xenia*’ collected during pre-harvest maturation (shadowed part, open circles) and postharvest storage period in a cold room at 2°C and RH~80-90% in normal atmosphere (green triangles). Time zero means the harvest time. Squares present shelf life points after predated storage in a cold room. Error bars are standard deviations. WSP – water soluble pectins, CSP – chelator (CDTA) soluble pectins, DASP – sodium carbonate soluble pectins.
