# Supplementary material for: The stiffening of the cell walls observed during physiological softening of pears
Source: Planta. 2015 Oct 26;243:519–29. doi: 10.1007/s00425-015-2423-0 (PMC4722064; doi:10.1007/s00425-015-2423-0)
Supplement: Supplementary file 2 — Supplementary material 2 (PDF 223 kb) [file 425_2015_2423_MOESM2_ESM.pdf]

The stiffening of the cell walls observed during physiological softening of pears

Planta

Artur Zdunek[1], Arkadiusz Kozioł[1], Justyna Cybulska[1], Małgorzata Lekka[2], Piotr M.Pieczywek[1]*

[1]Institute of Agrophysics, Polish Academy of Sciences, Doświadczalna 4, 20-290 Lublin, Poland,
[2]The Henryk Niewodniczański Institute of Nuclear Physics, Polish Academy of Sciences, Radzikowskiego 152, 31-342 Kraków, Poland
***corresponding author** a.zdunek@ipan.lublin.pl

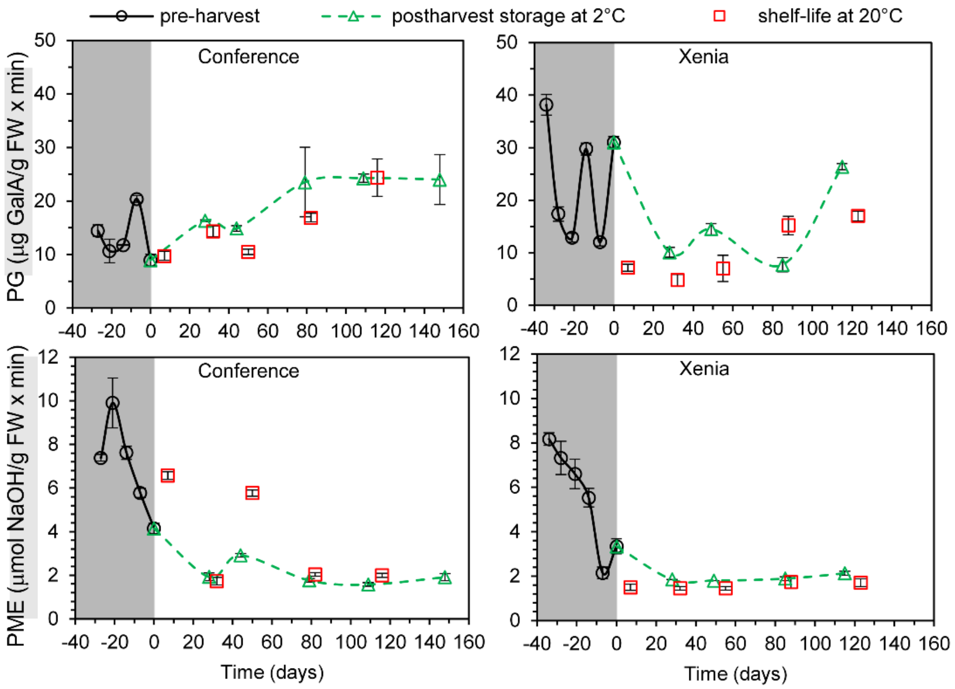

ESM_2. Polygalacturonase (PG) and pectin methylesterase (PME) activity in the studied pears ('*Xenia*' and '*Conference*') during pre-harvest maturation (shadowed part, open circles) and postharvest storage period in a cold room at 2°C and RH~80-90% in normal atmosphere (green triangles). Time zero means the harvest time. Squares present shelf life points after predated storage in a cold room. Error bars are standard deviations.
